# Polygenic risk prediction models for colorectal cancer: a systematic review

**DOI:** 10.1186/s12885-021-09143-2

**Published:** 2022-01-15

**Authors:** Michele Sassano, Marco Mariani, Gianluigi Quaranta, Roberta Pastorino, Stefania Boccia

**Affiliations:** 1 Section of Hygiene, University Department of Life Sciences and Public Health, Università Cattolica del Sacro Cuore, 00168 Roma, Italy; 2 Department of Woman and Child Health and Public Health - Public Health Area, Fondazione Policlinico Universitario A. Gemelli IRCCS, Roma, Italy

**Keywords:** Colorectal cancer, Prediction models, Single nucleotide polymorphisms, Genetic risk score, Polygenic, Meta-analysis

## Abstract

**Background:**

Risk prediction models incorporating single nucleotide polymorphisms (SNPs) could lead to individualized prevention of colorectal cancer (CRC). However, the added value of incorporating SNPs into models with only traditional risk factors is still not clear. Hence, our primary aim was to summarize literature on risk prediction models including genetic variants for CRC, while our secondary aim was to evaluate the improvement of discriminatory accuracy when adding SNPs to a prediction model with only traditional risk factors.

**Methods:**

We conducted a systematic review on prediction models incorporating multiple SNPs for CRC risk prediction. We tested whether a significant trend in the increase of Area Under Curve (AUC) according to the number of SNPs could be observed, and estimated the correlation between AUC improvement and number of SNPs. We estimated pooled AUC improvement for SNP-enhanced models compared with non-SNP-enhanced models using random effects meta-analysis, and conducted meta-regression to investigate the association of specific factors with AUC improvement.

**Results:**

We included 33 studies, 78.79% using genetic risk scores to combine genetic data. We found no significant trend in AUC improvement according to the number of SNPs (*p* for trend = 0.774), and no correlation between the number of SNPs and AUC improvement (*p* = 0.695). Pooled AUC improvement was 0.040 (95% CI: 0.035, 0.045), and the number of cases in the study and the AUC of the starting model were inversely associated with AUC improvement obtained when adding SNPs to a prediction model. In addition, models constructed in Asian individuals achieved better AUC improvement with the incorporation of SNPs compared with those developed among individuals of European ancestry.

**Conclusions:**

Though not conclusive, our results provide insights on factors influencing discriminatory accuracy of SNP-enhanced models. Genetic variants might be useful to inform stratified CRC screening in the future, but further research is needed.

**Supplementary Information:**

The online version contains supplementary material available at 10.1186/s12885-021-09143-2.

## Introduction

Colorectal cancer (CRC) is currently the third most commonly diagnosed type of cancer and the second cause of cancer death worldwide, with an estimated 1.8 million new cases and 880 thousands deaths in 2018, with a greater burden among males respect to females [1]. Typically, CRC can be considered a disease related to wealth. National levels of both CRC incidence and mortality are closely related to the income and development level of the country, with a cumulative risk of CRC or CRC death three times higher in countries with a high Human Development Index (HDI) than countries with a medium or low HDI [1].

Over the last decade, the majority of the countries in Europe, Oceania and North America witnessed a decrease in CRC mortality [2]. Likely, one of the main reasons for such a reduction in mortality rates in Western or developed countries could be related to the adoption of screening programs for CRC. As for CRC screening, different methods and strategies are effective at reducing its mortality and have been implemented in different countries worldwide, the most represented by fecal occult blood testing and fecal immunochemical test [3–6]. However, in recent years researchers have explored the possibilities of stratified screening, through the use of prediction models that could guide CRC risk assessment for asymptomatic patients [7]. In particular, most recent research in this field has focused on the inclusion of genetic factors into prediction models, particularly through the use of a genetic risk score (GRS) or a polygenic risk score (PRS) [8]. Furthermore, the increasing number of genome-wide association studies (GWASs) that are being conducted, with more than 70 GWASs currently published for CRC [9], is leading to a progressive improvement of our knowledge regarding the impact of common genetic variants or single nucleotide polymorphisms (SNPs) on the risk of CRC. In this sense, it should be noted that up to 35% of inter-individual variability in CRC risk has been attributed to genetic factors [10, 11], thus making the importance of this field for public health evident. Genetic factors could guide CRC risk assessment, thus improving the effectiveness of currently available screening strategies.

However, the methods currently used by researchers to incorporate genetic factors into prediction models for CRC and the characteristics of the latter are highly heterogeneous [8]. In addition, the potential improvement in discriminatory accuracy yielded by the addition of genetic factors to CRC prediction models including only traditional risk factors is still unclear, as it is not certain whether the number of genetic variants included in the models are related to such improvement.

For these reasons, the primary aim of the present study is to perform a systematic review regarding polygenic risk prediction models for CRC in order to identify which prediction models including genetic risk variants for CRC have been reported in the Scientific Literature.

The secondary aim is to assess the impact, in terms of improvement in discriminatory accuracy, of the addition of SNPs into prediction models with only traditional risk factors, and to test whether there is any relation between the number of SNPs included in the models and the improvement of their discriminatory accuracy. In addition, we aimed to evaluate which factors, besides the number of SNPs, influence the improvement of discriminatory accuracy.

## Methods and materials

We registered a protocol for this review on PROSPERO (Record ID: CRD42019135304), the international prospective register of systematic reviews. We uploaded on the PROSPERO register, prior to completing data extraction, the review title, timescale, team details, methods, and general information.

### Search strategy and study selection

We queried Pubmed, Web of Knowledge, Embase and CINAHL Complete electronic databases up to February 2020 using the elements of the Population, Intervention, Comparator, Outcome (PICO) model (P, population/patient; I, intervention/indicator; C, comparator/control; and O, outcome) [12]. In detail, our study population was represented by colorectal cancer; the intervention by SNPs; the comparator was none, and outcome was represented by risk prediction models. For this reason the following search string was built: (“Colorectal Neoplasms”[Mesh] OR “colorectal cancer” OR “colon cancer”) AND (“genetic variant” OR “genetic variants” OR “genetic variation” OR “genetic data” OR polymorphism OR SNP OR SNPs OR polygenic) AND (“risk stratification” OR “risk model” OR “risk profile” OR “risk profiling” OR “risk prediction” OR “risk determination” OR “risk discrimination” OR “risk score” OR “predictive model” OR “prediction model” OR “prediction models” OR “stratified screening”). The search was refined by hand searching and analysis of bibliographic citations in order to identify missing articles. No publication time limits were applied.

The manuscript was written following the recommendations of the Preferred Reporting Items for Systematic Reviews and Meta-Analyses (PRISMA) statement (Supplementary material) [13].

We systematically searched databases to retrieve all eligible scientific studies that developed, compared or validated a prediction model (or clinical prediction rule based on a model) using multiple (at least two) SNPs to predict the risk of CRC.

Two independent investigators (M.M. and M.S.) screened titles and abstracts of all potentially pertinent articles to identify eligible studies. We obtained, read and included, if relevant, full papers following the same procedures. At all levels, any discrepancies and disagreement were solved by consensus or by involving a third investigator (R.P.).

We included English-written peer-reviewed papers focusing on sporadic CRC reporting primary data and that evaluated the combined effect of two or more genes on CRC risk (e.g. GRS or PRS) or that reported a formal prediction model using genetic factors.

We excluded all studies that tested a model on simulated populations, pediatric populations, or dealing with inherited forms of colorectal cancer (e.g. Lynch syndrome). Furthermore, we did not include in this review commentaries, editorials, review papers, case reports, case series, book chapters, and articles with no primary data. Lastly, as for articles updating previous ones, we included only the last updated study.

### Data extraction

Data extraction was conducted independently by two researchers (M.M. and M.S.), for articles deemed relevant, using an in-depth piloted data extraction form and following an adapted version of the “CHecklist for critical Appraisal and data extraction for systematic Reviews of prediction Modelling Studies” (CHARMS) checklist [14]. Disagreements were solved through discussion or referral to a third reviewer (R.P.).

Extracted data include information regarding: author details; year of publication; study design; study population; sample size; genetic factors analyzed; GRS and related methods used to calculate it; factors other than genetic included in the model; internal and external validation; Area Under Curve (AUC) of non-SNP-enhanced models; AUC of SNP-enhanced models; Integrated discrimination improvement (IDI); and net reclassification improvement (NRI). In particular, NRI and IDI are measures used to compare the performances of two models, specifically an old model and a new model resulting from the addition of one or more predictors to the old one. The AUC is a measure of discriminatory accuracy and quantifies the ability of the model to discriminate between individuals with and without the outcome of interest [15], while NRI quantifies the ability of the new model to reclassify individuals compared to the previous one [16, 17], and IDI represents the difference in discrimination slopes of the new and the previous models, with the discrimination slope being the absolute difference in the averages of estimated probabilities of the event between those who experienced the event and those who did not [17–19].

For studies including both individuals with adenomas and CRC, we only extracted information about results related to CRC.

### Quality assessment

The risk of bias of included studies was assessed by two investigators (M.M. and M.S.) using the Prediction model Risk Of Bias ASsessment Tool (PROBAST) [20]. PROBAST is a tool developed to assess the risk of bias and applicability of prediction model studies and contains a total of 20 signaling questions divided into 4 key domains that regard: participants, predictors, outcome, and analysis. Each domain is rated for risk of bias (low, high or unclear risk of bias). The signaling questions can be rated as “yes”, “probably yes”, “probably no”, “no” or “no information”. Every signaling question is phrased so that “yes” or “probably yes” mean absence of bias, while “no” or “probably no” warn for potential risk of bias. The first three domains that regard participants, predictors and outcome are also assessed for concerns for applicability (high, low, or unclear) to the defined review question.

### Statistical analysis

Statistical analysis was carried out including only studies that reported both a model with only traditional risk factors and one incorporating also genetic factors. For studies that calculated the AUCs of the same model constructed in different ways (e.g. counted GRS and weighted GRS), only the model showing the best performance or, for those showing the same values of AUC, the simplest one was included in the analysis. Stratification according to the number of SNPs was conducted using tertiles based on the distribution of the number of SNPs included in the models across included studies, with lowest, mid, and highest tertile being represented by ≤22, 23–47, and ≥ 48 SNPs, respectively. We calculated standard errors of AUCs using the Hanley and McNeil method [15].

First, we tested whether a significant trend in the increase of the AUC of the SNP-enhanced models according to the number of SNPs included in the models could be observed. Secondly, we estimated the Pearson’s correlation coefficient between AUC improvement and number of SNPs. Eventually, we investigated whether the increasing number of SNPs added to the baseline models determined an observable trend in the improvement of the AUC by drawing a forest plot. In order to calculate a pooled AUC improvement for SNP-enhanced models compared with non-SNP-enhanced models, we conducted a meta-analysis using the random effects model, based on the assumption that clinical and methodological heterogeneity was very likely to occur and to have an effect on the results. We quantified statistical inconsistency using the *I*^*2*^ statistic. Moreover, we assessed whether specific factors (number of cases, number of SNPs, publication year, AUC of non-SNP-enhanced model, ethnicity of study participants, number of traditional risk factors in the model, and inclusion of gender in the model both as a covariate or by stratification) were significantly associated with AUC improvement and explained statistical heterogeneity by conducting meta-regression, with *p*-values adjusted for multiple testing computed using 1000 Monte-Carlo permutations.

All statistical analyses were conducted using the Stata software version 13.0 [21].

## Results

### Study selection

**Fig. 1:**
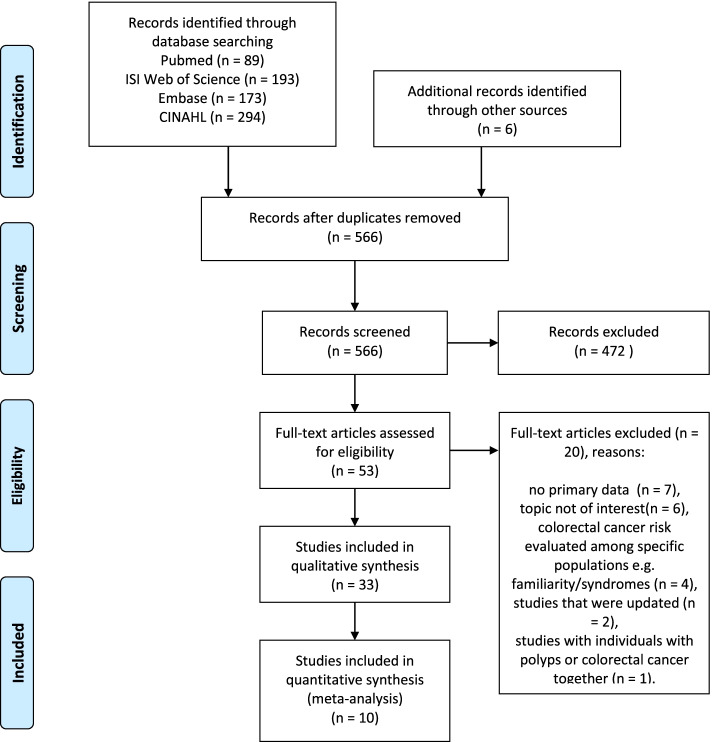
PRISMA flow-chart of the study selection process

The results of abstract and full-text screening with reasons for exclusion are shown in the PRISMA flow diagram [13] in Fig. 1. The database research resulted in 749 records. A total of 6 articles were retrieved through hand search. After checking for duplicates, 566 articles were analyzed for eligibility and 472 were excluded after title and abstract screening. The remaining 94 articles were selected for full-text review, resulting in 33 articles included in the qualitative synthesis and 10, eventually, included in the meta-analysis. The main causes for exclusion were represented by: articles with no primary data or with simulated populations (35%), non-pertinent articles (30%); articles with population represented by individuals with inherited forms of colorectal cancer (20%); eventually, studies that were later updated and published (10%) or that gathered together with CRC cancer and colorectal benign polyps without distinguishing these two populations (5%).

### Study and population characteristics

The main characteristics of the articles included in the systematic review are summarized in Table 1. Studies included in this review were published from 2008 and 2019. Most of them were case-control studies (78.79%) [22, 23, 25, 27–36, 39, 41–43, 45–47, 49–54], followed by 5 cohort studies (15.15%) [24, 38, 40, 44, 48], and 2 (6.06%) case-cohort studies [26, 37]. No sample overlap can be reported across studies. Twenty-one (63.64%) evaluated risk prediction models among individuals of European ancestry [23, 24, 26–28, 30–32, 34, 35, 38–46, 49, 50], 12 (36.36%) among a population of Asian ancestry [22, 25, 29, 33, 36, 37, 47, 48, 51–54]. Population sizes ranged from 603 [47] to 361,543 [44] individuals.

**Table 1:** Main characteristics of the included studies in the systematic review

First author, year [ref]	Study design	Study population	Number of study participants	Type of genetic variants used	GRS computation	Non-genetic factors included in the model	AUC (95% CI) of model without SNPs	AUC (95% CI) of SNP-enhanced model	IDI; NRI
Abe M, 2017 [22]	Case-control	Japanese	Derivation: 558 cases and 1116 controls; Replication: 547 cases and 547 controls.	11 SNPs (6 derived from GWASs in US/Europeans, 5 identified in GWASs in East Asians)	Unweighted GRS			Derivation study: 0.6392; Replication study: 0.5695	
Balavarca Y, 2019 [23]	Case-control	German	236 non-advanced adenomas, 291 advanced CRC; 487 controls	39 SNPs	Unweighted GRS; Weighted GRS using weights derived from the same study	Gender, age, FH of CRC, smoking, alcohol intake, red meat consumption, use of NSAIDs, previous colonoscopy and polyps history	0.584 (0.545–0.622)	Unweighted GRS: 0.636 (0.599–0.672); Weighted GRS: 0.616 (0.579–0.654)	
Chandler PD, 2018 [24]	Cohort	US	23,294 individuals, 329 CRC cases	5 SNPs	Unweighted GRS				
Cho YA, 2019 [25]	Case-control	Korean	632 cases 1295 controls	13 SNPs	Unweighted GRS; Weighted GRS using weights derived from the same study	BMI, physical activity, diet, smoking, alcohol consumption.			
de Kort S, 2019 [26]	Case-cohort	Dutch	1907 CRC cases, 2729 subcohort members	18 SNPs	Unweighted GRS	Age, BMI, pant size, CRC first degree relative, smoking, nonoccupational physical activity, intake of: alcohol, meat, vegetables, fish, sweets, added sugar, saturated fats and fiber, total energy.			
Dunlop MG, 2013 [27]	Case-control	European descendents	Genotypes alone: 39,266; In combination with other factors: 11,324; External validation case-control sets: 1563 Swedish cases and 1504 controls, 702 Finnish cases and 418 controls.	10 SNPs	Unweighted GRS	FH of CRC, age, gender.			
Hiraki LT, 2013 [28]	Case-control	European descendants	10,061 cases and 12,768 controls	4 SNPs	Unweighted GRS	Age, gender, center, smoking, batch effects, FH of CRC, BMI, NSAIDs use, alcohol use, dietary calcium, folate and red meat intake, sedentary status, hormone replacement therapy when possible and according to the study.			
Hosono S, 2016 [29]	Case-control	Japanese	Derivation set: 558 cases and 1116 controls Replication set: 547 cases and 547 controls	6 SNPs	Unweighted GRS	Age, smoke, alcohol consumption, folate intake, BMI, FH of CRC, physical activity.	Derivation study: 0.7009; Replication study: 0.5232	Derivation study: Genetic only risk score: 0.6046; Combined (genetic + traditional): 0.7167; Replication study: Genetic only: AUC 0.6391; Combined (genetic + traditional) AUC 0.6356	
Hsu L, 2015 [30]	Case-control	European descendants	Training set: 5811 cases and 6302 controls; Validation set: 866 cases and 869 controls.	27 SNPs	Unweighted GRS; Weighted GRS using weights derived from literature (results not reported)	Age, gender, FH of CRC, history of endoscopic examinations	Men 0.51 (0.48–0.53); Women 0.52 (0.50–0.55)	Men: AUC 0.59 (0.54–0.64); Women: 0.56 (0.51–0.61)	
Huyghe JR, 2019 [31]	Case-control	European descendants	1439 cases and 720 controls	95 SNPs	Weighted GRS using weights derived from the same study				
Ibáñez-Sanz G, 2017 [32]	Case-control	Spanish	1336 cases and 2744 controls.	21 SNPs	Unweighted GRS; Weighted GRS using weights derived from literature and from the same study (results not reported)	Alcohol consumption, BMI, physical activity, red meat and vegetables intake, NSAIDs/aspirin use, FH of CRC	Environmental risk factors and family history: 0.61 (0.59–0.64)	0.63 (0.60–0.66)	
Iwasaki M, 2017 [33]	Case-control	Japanese men	675 cases and 675 controls	6 SNPs	Weighted GRS using weights derived from the same study	Age, BMI, alcohol consumption, smoking.	0.60	0.66	Significant difference in the inclusive model with a GRS compared to the non-genetic model for the IDI (0.0052; 95% CI: 0.0023–0.0081), continuous NRI (0.36; 95% CI: 0.0023–0.71), and NRI (0.26; 95% CI: 0.0039–0.43).
Jenkins MA, 2019 [34]	Case-control	North American and Australian	1181 cases and 999 controls	45 SNPs	Weighted GRS using weights derived from literature	FH of CRC			
Jeon J, 2018 [35]	case-control	European descendants	Training set: 4875 cases and 5291 controls Validation set: 4873 cases and 5299 controls.	63 SNPs	Weighted GRS using weights derived from the same study	Gender, height, body mass index, education, type 2 diabetes mellitus, smoking status, alcohol consumption, NSAID/aspirin use, regular use of postmenopausal hormones, gender- and study-specific quartiles of smoking pack-years and dietary factors, total-energy, and physical activity	Men: 0.60 (0.59–0.61); Women: 0.60 (0.59–0.61)	Men: 0.63 (0.62–0.64); Women: 0.62 (0.61–0.63)	
Jo J, 2012 [36]	Case-control	Korean	187 cases and 976 controls	3 SNPs in men, 5 SNPs in women	Unweighted GRS; Weighted GRS using weights derived from the same study	FH of CRC, age.	Conventional risk factors alone, men: 0.692 (0.647–0.732); Conventional risk factors alone, women: 0.603 (0.569–0.637)	Counted GRS plus traditional risk factors, men: 0.729 (0.682–0.767); Weighted GRS plus traditional risk factors, men: 0.719 (0.677–0.761); Counted GRS plus traditional risk factors, women: 0.650 (0.615–0.680); Weighted GRS plus traditional risk factors: 0.646 (0.612–0.674)	
Jung KJ, 2015 [37]	Case-cohort	Korean	173 cases and 1514 controls	7 SNPs	Unweighted GRS; Weighted GRS using weights derived from the same study	TRS: age, gender, smoking status, fasting serum glucose, FH of CRC	0.73 (0.69–0.78)	0.74 (0.70–0.78)	The NRI (95% CI) for a prediction model with GRS compared to the model with TRS alone was 0.17 (− 0.05–0.37) for colorectal cancer, − 0.17 (− 0.33–0.21) for colon cancer, and 0.41 (0.10–0.68) for rectal cancer.
Jung SY, 2019 [38]	Cohort	European ancestry (women only)	6539 individuals, 472 cases developed CRC	54 SNPs		Age and % calories from saturated fatty acid			
Marshall KW, 2010 [39]	Case-control	North American	Training set: 112 CRC and 120 controls. Validation set: 202 CRC and 208 controls (only individuals aged ≥50 years).	7 genes				Training set: AUC 0.80 (0.74–0.85); Validation set: AUC 0.80 (0.76–0.84)	
Prizment AE, 2013 [40]	Cohort	Caucasian	8657 individuals (205 cases)	20 SNPs	Weighted GRS using weights derived from literature				
Rodriguez-Broadbent H, 2017 [41]	Case-control	European descendants	9254 cases and 18,386 controls	38 SNPs related to total cholesterol circulating levels, 14 SNPs related to triglyceride circulating levels, 9 SNPs related to LDL circulating levels, 43 SNPs related to HDL circulating levels					
Schmit SL, 2019 [42]	Case-control	European descendants	Discovery stage: 36,948 cases and 30,864 controls; Replication set: 12,952 cases and 48,383 controls; Generalizability in East Asians, African Americans, and Hispanics: 12,085 cases and 22,083 controls.	76 SNPs: 67 previously published SNPs and 9 novel SNPs	Weighted GRS using weights derived from the same study				
Shi Z, 2019 [43]	Case-control	Caucasian	387 cases and 13,427 controls	30 SNPs	Weighted GRS using weights derived from literature	Population-standardization			
Smith T, 2018 [44]	Cohort	UK	Taylor model: 361,543 (1623 cases); Wells model: 286,877 (1294 cases)	41 SNPs	Weighted GRS using weights derived from literature	Taylor model: age-specific CRC rates and estimated RR for different degrees of FH of CRC. Wells model: age, diabetes, multi-vitamin usage, FH of CRC, education, BMI, alcohol use, physical activity, NSAIDs use, red meat intake, smoking and estrogen use (women only).	Taylor model: 0.67 (0.65–0.68); Wells model: 0.68 (0.67–69)	Taylor model:0.69 (0.67–0.70); Wells model: 0.69 (0.65–0.68)	
Thrift AP, 2015 [45]	Case-control	European descendants	10,226 cases and 10,286 controls	696 SNPs	Weighted GRS using weights derived from literature				
Thrift AP, 2015 [46]	Case-control	European descendants	10,226 cases and 10,286 controls	77 SNPs for BMI; 47 SNPs for waist-hip ratio (WHR)	Weighted GRS using weights derived from literature				
Wang HM, 2013 [47]	Case-control	Taiwanese	218 cases and 385 controls	16 SNPs in the short model; 26 SNPs in the full model				16-SNPs model: 0.724; 26-SNPs model: 0.734	
Wang K, 2018 [48]	Cohort	Chinese	64 CRC cases (172 digestive cancer cases, 9636 controls)	9 SNPs			AFP level: 0.523 (0.456–0.591); CA19–9 level:0.524 (0.451–0.597); CEA level: 0.568 (0.492–0.645); AFP, CA19–9, CEA level: 0.509 (0.439–0.579)	AFP level -genetic corrected: 0.524 (0.458–0.591); CA19–genetic corrected CA19–9 level: 0.525 (0.452–0.597); CEA level-genetic corrected 0.572 (0.495–0.649); AFP, CA19–9, CEA level-genetic: 0.564 (0.487–0.641)	
Weigl K, 2018 [49]	Case-control	German	Genotype: 294 advanced neoplasms, 249 non-advanced adenomas, 500 controls Replication: 462 controls, 140 advanced adenomas, 355 non-advanced adenomas	48 SNPs (replication analyses within the TCPS with a subset of 35 SNPs of the original GRS)	Unweighted GRS; Weighted GRS using weights derived from literature (results not reported)	Gender, age, previous colonoscopy, physical activity, BMI	Model adjusted for age and gender: 0.599; Model adjusted for age, gender, previous colonoscopy, physical activity: 0.607; Model adjusted for age, gender, previous colonoscopy, physical activity, BMI: 0.615	Model adjusted for age and gender: 0.653; Model adjusted for age, gender, previous colonoscopy, physical activity: 0.658; Model adjusted for age, gender, previous colonoscopy, physical activity, BMI: 0.665	The NRI and IDI of model including Genetic Risk Score were respectively of 0.29 (0.14–0.43) and 0.04 (0.03–0.05) when the model was adjusted for age and gender; 0.30 (0.15–0.44) and 0.04 (0.03–0.05) when adjusted for age, gender, previous colonoscopy, physical activity and 0.29 (0.14–0.43) and 0.04 (0.03–0.05) when the model was adjusted for age, gender, previous colonoscopy, physical activity, BMI.
Weigl K, 2018 [50]	Case-control	German	2363 cases and 2198 controls.	44 SNPs	Unweighted GRS; Weighted GRS using weights derived from literature (results not reported)	Gender, age, education, previous colonoscopy, smoking, hormone replacement therapy (women only), BMI, FH of CRC			
Xin J, 2018 [51]	Case-control	Chinese	1316 cases and 2229 controls	14 SNPs	Unweighted GRS; Weighted GRS using weights derived from literature and from the same study	Smoking status	The highest quartile respect to the lower quartile showed an OR (95%CI) of: 2.70 (2.06–3.54) in the simple count GRS model, 2.74 (2.19–3.43) in the directed logistic regression GRS model, 2.56 (2.05–3.20) in the odds ratio weighted GRS model, 2.90 (2.32–3.63) in the explained variance weighted GRS model, 2.51 (2.01–3.14) in the explained variance weighted OR GRS model.		Model were compared among each other respect to NRI (95%CI; *p*-value) and IDI (95%CI; *p*-value): the simple-count-GRS vs. logistic regression weighted OR-GRS showed an NRI of − 0.082 (− 0.159, − 0.007; *p* value: 0.033) and an IDI of − 0.002 (− 0.004, − 2.33E− 04; 0.028); the simple-count-GRS vs. explained variance weighted OR-GRS showed an NRI of 0.017 (− 0.055, 0.090; 0.638) and an IDI of 2.80E− 04 (− 0.001, 0.001; 0.567); logistic regression weighted-GRS vs. explained variance weighted OR-GRS showed an NRI − 0.077 (− 0.153, − 0.001; 0.046) and an IDI of − 5.54E− 04 (− 0.001, − 3.17E− 05; 0.038). In addition, a model including only smoking factors was with a model including smoking factors and simple count GRS (SC-GRS), with an increased AUC, NRI and IDI in combined model of 0.084, 0.317 (0.225, 0.408) and 0.031 (0.023, 0.039)
Xin J, 2019 [52]	Case-control	Chinese	Chinese studies: 2248 cases and 3173 controls; GECCO study: 4461 cases and 4140 controls	Chinese studies: 19 SNPs vs. 58 SNPs; GECCO study: 19 SNPs vs. 75 SNPs	Weighted GRS using weights derived from the same study	Gender, age, first principal component		Chinese studies: 19 SNPs model of 0.597 (0.581–0.613), 58 SNPs model of 0.623 (0.604–0.642); GECCO study: 19 SNPs model of 0.575 (0.563–0.587), 58 SNPs model of 0.585 (0.573–0.597)	
Yeh CC, 2007 [53]	Case-control	Taiwanese	727 cases and 736 controls	10 SNPs		Age, education, physical activity, coffee consumption, cigarette consumption, alcohol use, staple consumption, meat, vegetable/fruit and fish/shrimp intake.			
Zhang L, 2017 [54]	Case-control	Chinese	369 cases and 929 controls	4 SNPs		Age, BMI, physical activity, emotion status, mental stress, cholesterol, drinking and smoking, vegetables and seafood consumption			

*CRC* colorectal cancer, *SNP* single nucleotide polymorphism, *ERS* environmental risk score, *GRS* genetic risk score, *TRS* traditional risk score, *PRS* polygenic risk score, *ct-DNA* circulating tumor-DNA, *RR* relative risk, *HR* hazard ratio, *OR* odds ratio, *GWAS* genome-wide association study, *BMI* body mass index, *FH* family history, *NSAID* nonsteroidal anti-inflammatory drug

### Risk prediction models characteristics

The number of genetic variants evaluated in the risk prediction model ranged from 4 [54] to 696 SNPs [45]. A complete list of SNPs included in each study is provided in Table S1.

In order to include genetic factors into prediction models, different methodologies were investigated across the included studies. In particular, 26 (78.79%) studies used a GRS, 11 (42.31%) of which used a weighted GRS [31, 33–35, 40, 42–46, 52], other 6 (23.08%) studies used an unweighted GRS [22, 24, 26–29]. Instead, a total of 9 studies (34.62%) used both unweighted and weighted methods to develop risk scores [23, 25, 30, 32, 36, 37, 49–51].

Of the remaining 7 studies that did not use GRS (21.21%), one [39] derived 7 genes from a larger set. After gene profiling and cluster analysis, specific genes were selected, further validated and evaluated for predictive performance. The second one performed a Mendelian randomization analysis to assess the association between hyperlipidemia and CRC using Burgess statistics [55] and a fixed-effects meta-analysis to derive final odds ratios [41], while another one [47] applied logistic regression, Jackknife feature selection and ANOVA testing to construct the prediction model. Other authors [53] applied a stepwise selection procedure in order to determine the inclusion or exclusion of the putative risk factors from the models, and the combined effect of genes on colorectal cancer risk was assessed by multivariate unconditional logistic regression. Instead, 2 studies used machine learning approaches [38, 54]; the last one evaluated the predictive accuracy of genetic corrected serum levels of specific biomarkers compared to uncorrected ones [48].

### Difference in discriminatory accuracy between SNP-enhanced and traditional risk factor models

Using the Swets classification [56], i.e. low accuracy when the AUC is between 0.5 and 0.7, moderate accuracy between 0.7 and 0.9, only two of the studies that included both a traditional risk factor only model and one incorporating also genetic factors found a moderate discriminatory accuracy. The first study [36] showed that, only among males, AUC values for models including counted GRS and weighted GRS reached 0.729 (95% CI: 0.682, 0.767) and 0.719 (95% CI: 0.677, 0.761), respectively; while models without SNPs showed low accuracy (i.e. AUC lower than 0.7). The second study [37] found moderate discriminatory accuracy for both SNP and non-SNP-enhanced models. In particular when overall colon and rectal cancer risk, colon cancer risk only, and rectal cancer risk only were separately considered, SNP-enhanced models yielded AUC values of 0.74 (95% CI: 0.70, 0.78), 0.75 (95% CI: 0.69, 0.81), and 0.74 (95% CI: 0.68, 0.79), respectively; while non-SNP-enhanced model yielded AUC values of 0.73 (95% CI: 0.69, 0.78), 0.76 (95% CI: 0.70, 0.83), and 0.71 (95% CI: 0.65, 0.77), respectively.

A total of 4 articles [33, 37, 49, 51] used the NRI and/or the IDI to compare the performances of two models (traditional only vs genetic enhanced model). In the first article [37], the NRI for a prediction model with GRS respect to the traditional risk score model was 0.17 (95% CI: − 0.05, 0.37) for CRC, − 0.17 (95% CI: − 0.33, 0.21) for colon cancer only, and 0.41 (95% CI: 0.10, 0.68) for rectal cancer only. The second one [33] found an increase in the inclusive model compared to the non-genetic model for the mean IDI (0.015) and the mean continuous NRI (0.39). After defining risk categories of NRI by arbitrary cut-off values of 1.5 and 3% of 10-year absolute risk of developing colorectal cancer, the mean NRI value was equal to 0.12 when the non-genetic and inclusive models were compared. The third [49] showed an increase in the NRI in all the models when different variables were included in the model (Table 1). Eventually, the last one [51] found that the traditional model with smoking status showed worse performance respect to the combined model that included genetic (simple count GRS,) and smoking factors: NRI of 0.317 (95% CI: 0.225, 0.408) and IDI of 0.031 (95% CI: 0.023, 0.039).

### AUC analysis

A total of 14 risk prediction models, from 10 studies were included in the AUC analysis [23, 30, 32, 33, 35–37, 44, 49, 51]. We found no significant trend regarding the increase in the AUC of the SNP-enhanced risk prediction models according to the number of SNPs included in the models and, when the AUC was tested for trend, no significant association was retrieved (*p* for trend = 0.774). Pearson’s correlation coefficient between AUC improvement and number of SNPs was also estimated, r = − 0.0993 (95% CI: − 0.541, 0.385; *p* = 0.6951). No correlation could be found between the number of SNPs and AUC increase.

**Fig. 2:**
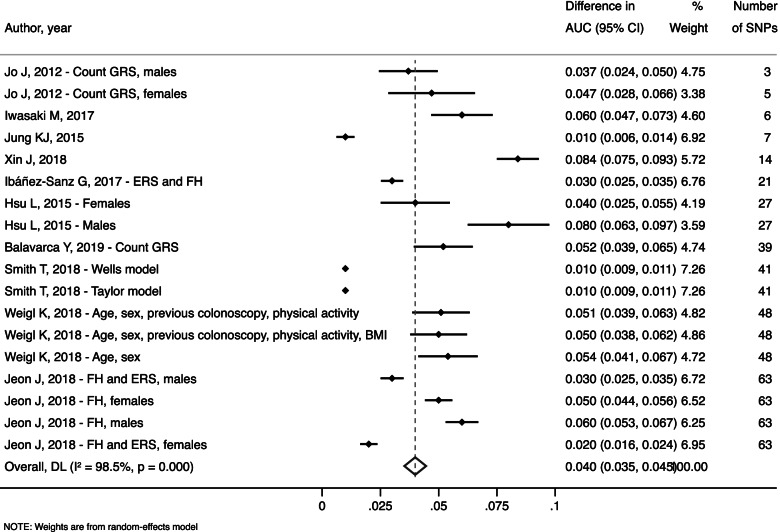
Overall improvement in AUC for SNP-enhanced prediction models compared with non-SNP-enhanced models

The meta-analysis resulted in a pooled estimate of AUC improvement for SNP-enhanced prediction models compared with non-SNP-enhanced models of 0.040 (95% CI: 0.035, 0.045) for all 14 models (Fig. 2). High heterogeneity was found reaching 98.5% (*p* < 0.001).

**Fig. 3:**
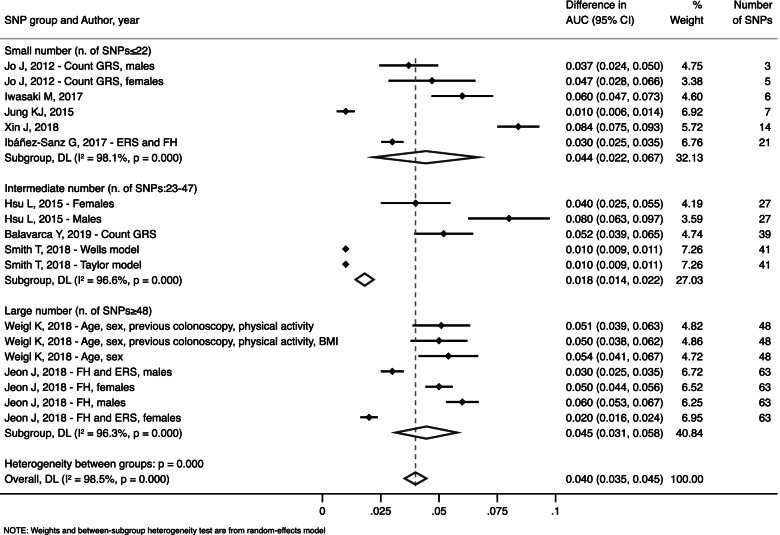
Improvement in AUC for SNP-enhanced prediction models compared with non-SNP-enhanced models stratified by the tertile of number of SNPs included in the model

A stratified analysis by number of SNPs included across models was performed (Fig. 3). The AUC difference between the SNPs-enhanced models respect to non-SNP-enhanced models for the lowest tertile of SNPs added to the model (less than or equal to 22 SNPs) resulted in an improvement of 0.044 (95% CI: 0.022, 0.067). As to the mid (23–47 SNPs) and highest tertiles (more than or equal to 48 SNPs) of SNPs added, the estimates showed an improvement in the AUC of 0.018 (95% CI: 0.014, 0.022) and 0.045 (95% CI: 0.031, 0.058), respectively.

The results of the meta-regression (Table 2) showed that the factor more strongly associated, inversely, with AUC improvement after the addition of SNPs to a model with only traditional risk factors was the AUC of the non-SNP-enhanced model (*p* < 0.001). Furthermore, an inverse significant association was found also between the number of cases included in the study and AUC improvement (*p* = 0.002). Eventually, ethnicity was associated with AUC improvement too (*p* = 0.023), with better AUC improvements achieved by models constructed among Asians compared with individuals with European ancestry. No significant associations were found for other investigated factors. Overall, the factors included in the meta-regression explained almost half statistical heterogeneity, with a residual *I*^*2*^ equal to 54.18%.

**Table 2:** Results of the meta-regression assessing which factors are associated with AUC improvement of SNP-enhanced models compared with non-SNP enhanced models

	Coefficient	95% Confidence Interval	***p***-value	Adjusted ***p***-value
Number of cases	−0.000016	−0.0000243, − 7.63*10^− 6^	0.002	0.027
Number of SNPs	0.0004986	0.0000216, 0.0009757	0.042	0.170
Year of publication	0.0021238	−0.0012521, 0.0054998	0.191	0.468
AUC of non-SNP enhanced model	−0.3485498	−0.4171094, − 0.2799903	< 0.001	< 0.001
Ethnicity (Asian vs European)	0.0313164	0.0151622, 0.0474705	0.002	0.023
Number of traditional risk factors in the model	−0.0000322	−0.0010623, 0.0009979	0.946	1.000
Gender considered in the construction of the model	−0.0086505	−0.0191019, 0.001801	0.095	0.316

*SNP* single nucleotide polymorphism

### Quality assessment

Results of the overall risk of bias and applicability assessment can be found in Table 3.

**Table 3:** Results of the risk of bias for each domain of the PROBAST tool

First author, year [ref]	Risk of bias (ROB)								Applicability						Overall	
	Participants		Predictors		Outcome		Analysis		Participants		Predictors		Outcome		Risk of Bias	Applicability
	Dev	Val	Dev	Val	Dev	Val	Dev	Val	Dev	Val	Dev	Val	Dev	Val		
Abe M, 2017 [22]	High	High	High	High	Unclear	Unclear	High	High	Low	Low	Low	Low	Low	Low	High	Low
Balavarca Y, 2019 [23]	High		High		Low		High		High		Low		Low		High	High
Chandler PD, 2018 [24]	Low		High		High		High		Low		Low		Low		High	Low
Cho YA, 2019 [25]	High		High		High		High		Low		Low		Low		High	Low
de Kort S, 2019 [26]	Low		High		Low		High		Low		Low		Low		High	Low
Dunlop MG, 2013 [27]	High	High	Unclear	Unclear	Unclear	Unclear	Low	Low	Low	Low	Low	Low	Low	Low	High	Low
Hiraki LT, 2013 [28]	High		High		High		High		Low		Low		Low		High	Low
Hosono S, 2016 [29]	High	High	High	High	Unclear	Unclear	High	High	Low	Low	Low	Low	Low	Low	High	Low
Hsu L, 2015 [30]	High	Low	Low	Low	Low	Low	Unclear	Unclear	Low	Low	Low	Low	Low	Low	High	Low
Huyghe JR, 2019 [31]	Low		Low		Low		Unclear		Low		Low		Low		Unclear	Low
Ibáñez-Sanz G, 2017 [32]	High		Unclear		Low		Unclear		Low		Low		Low		High	Low
Iwasaki M, 2017 [33]	Low		Unclear		Low		Unclear		Low		Low		Low		High*	Low
Jenkins MA, 2019 [34]		High		Low		High		Unclear		Low		Low		Low	High	Low
Jeon J, 2018 [35]	High	High	Low	Low	Low	Low	Unclear	Unclear	Low	Low	Low	Low	Low	Low	High	Low
Jo J, 2012 [36]	Low		Unclear		Low		High		Low		Low		Low		High	Low
Jung KJ, 2015 [37]	Low		Unclear		Low		High		Low		Low		Low		High	Low
Jung SY, 2019 [38]	Low		High		Unclear		High		High		High		Low		High	High
Marshall KW, 2010 [39]	High	High	Unclear	Unclear	Low	Low	High	High	Unclear	Unclear	Low	Low	Low	Low	High	Unclear
Prizment AE, 2013 [40]	Low		Low		Low		High		High		Low		Low		High	High
Rodriguez-Broadbent H, 2017 [41]	High		High		High		High		High		Low		Low		High	High
Schmit SL, 2019 [42]	High	High	Unclear	Unclear	Low	Low	Unclear	Unclear	Low	Low	Low	Low	Low	Low	High	Low
Shi Z, 2019 [43]	Low		Low		Low		Unclear		Low		Low		Low		Unclear	Low
Smith T, 2018 [44]		Low		Low		Unclear		High		Low		Low		Low	High	Low
Thrift AP, 2015 [45]	High		High		High		High		High		Low		Low		High	Low
Thrift AP, 2015 [46]	High		High		High		High		High		Low		Low		High	Low
Wang HM, 2013 [47]	High		Unclear		Low		High		Unclear		Low		Low		High	Unclear
Wang K, 2018 [48]	Low		Low		Low		High		Low		Low		Low		High	Low
Weigl K, 2018 [49]	High	High	Unclear	Unclear	Low	Low	High	High	High	High	Low	Low	Low	Low	High	High
Weigl K, 2018 [50]	High		Unclear		Low		High		Low		Low		Low		High	Low
Xin J, 2018 ^a^ [51]		Low		Unclear		Unclear		High		Low		Low		High	High	High
Xin J, 2019 [52]	High		Unclear		Low		Unclear		Low		Low		Low		High	Low
Yeh CC, 2007 [53]	High		Unclear		Low		High		Low		Low		Low		High	Low
Zhang L, 2017 [54]	High		Unclear		Unclear		High		Low		Low		Low		High	Low

In the risk of bias assessment, “low” means low risk of bias, “high” means high risk of bias, and “unclear” means it was not possible to assess the risk of bias. In the applicability section, “high” means high concern for applicability, “low” means low concern for applicability, and “unclear” means it was not possible to assess the applicability. Risk of bias assessed with the PROBAST tool

* = a high risk of bias was assigned because of the lack of external validation, among other reasons

^a^ = quality assessment conducted only for the validation phase of the study, since model development involved a simulated population (among our exclusion criteria)

The majority of the studies (93.94%) were scored as having high risk of bias [22–30, 32–42, 44–54, 57], 2 (6.06%) studies were rated as having an overall unclear risk of bias [31, 43].

A total of 22 (66.67%) studies were assessed only for the development of the model, 8 (24.24%) studies were assessed for both model development and validation, 3 (9.09%) only for model validation.

As to the model development, 66.67, 36.67, 20.00 and 70.00% of the studies were assessed as having high risk of bias respect to participants, predictors, outcome and statistical analysis, respectively; 33.33, 20.00, 63.33, 3.33% were deemed as having a low risk of bias, while 0.00, 43.33, 16.67, 26.67% were assessed as having unclear risk of bias respectively for participants, predictors, outcome and statistical analysis assessment.

As to validation models, 27.27, 36.36, 45.45, 9.09% of the included studies were assessed as having low risk of bias for participants, predictors, outcome and statistical analysis, respectively; while 72.73, 63.64, 54.55 and 90.91% were rated as high or unclear risk of bias.

Regarding the applicability of prediction models, in development model studies 30.00, 3.33, and 0.00% were at high or unclear risk; in validation studies 18.18, 0.00, 9.09% were at high or unclear risk as to, respectively, participants, predictors and outcome.

## Discussion

Overall, from the 35 studies that we included in our systematic review we identified prediction models for CRC incorporating genetic factors, with extreme heterogeneity regarding the number of genetic factors included. Instead, as for the methods to include genetic factors in the prediction model, most studies used a weighted GRS, with a minority of them using either the count model or both the weighted and count methods.

As for studies reporting the AUC value of the model, most of them could not find a satisfactory discriminatory accuracy (e.g. AUC > 0.7 [56]) for their models, even though the addition of genetic factors to traditional risk factors improved it, with an improvement in the AUC ranging from 0.010 [37, 44] to 0.084 [51]. Nonetheless, similarly to what was previously reported for breast cancer [58], we found no evidence of association or correlation between the number of SNPs included in the model and the improvement in the AUC value. However, among studies comparing two or more models, only a minority reported data on NRI or IDI, witnessing the need to better quantify and report the improvement of accuracy of a model when adding new biomarkers or genetic data [59]. According to the interpretation suggested by Pencina et al. for NRI values, all these four studies showed a weak or intermediate strength of SNPs (for all of them in the form of a GRS), in terms of discriminatory potential, when added to models with only traditional risk factors [17].

Regarding the pooled improvement in AUC, a clear trend in the improvement of AUC related to the number of SNPs could not be found. The best results were achieved in the lowest (≤22 SNPs) and highest (≥48 SNPs) tertiles of SNPs incorporated into the models, which led to a larger improvement in AUC compared with the mid tertile (23–47 SNPs). As expected, due to the extremely high heterogeneity among variables, regarding various SNPs and several environmental factors included in the retrieved prediction models and among statistical methods used to incorporate such variables in the models, our meta-analysis results show significant statistical heterogeneity, witnessed by the high values of the I^2^ obtained. For this reason, the results of our study should be interpreted cautiously and cannot be considered conclusive.

Similarly to our results, Fung et al. reported that the addition of genetic information improved discriminatory accuracy of the identified prediction models for breast cancer, even though AUC improvement was found to be not correlated or associated with the number of SNPs that were included in the model [58].

It should be noted that the improvement of AUC values with the addition of biomarkers, such as SNPs, to a model depends on the starting AUC value, which means the higher the AUC value of the model including only traditional risk factors, the smaller the improvement in AUC after adding genetic information into the model [17, 60, 61]. This was further confirmed by the results of our meta-regression. In addition, an inverse relation with AUC improvement was found also for the number of cases included in the study, which could actually be linked to the AUC of the non-SNP enhanced model. Likely, the higher the number of cases in the study, the larger the AUC of the non-SNP enhanced model and, hence, the smaller the AUC improvement.

Furthermore, the ethnicity of study participants was found to significantly affect AUC improvement, suggesting possible differences in the role of genetic factors between different populations, and witnessing the need to foster research in the field of genetic prediction models for all ethnicities [62]. The distribution of genetic factors associated with a specific cancer may vary between different ethnicities even more than traditional risk factors, thus the need for ethnicity-specific genome-wide association studies (GWAS) is crucial to inform the development of specific prediction models for different ethnicities [22, 63]. Furthermore, the importance of the chosen population in the construction of predictive models should be properly taken into account, as a model is applicable only to the specific population it was designed for [60].

Eventually, results of the meta-regression showed that the number of SNPs, publication year, the number of traditional risk factors in the model, and inclusion of gender in the model were not associated with AUC improvement. However, they largely explained statistical heterogeneity between included studies.

As far as we know, previous systematic reviews on prediction models for CRC including genetic factors were limited to a qualitative synthesis [8]. Hence, to our knowledge, our study is the first to investigate, through a quantitative approach, the improvement in discriminatory accuracy that can be obtained through the incorporation of SNPs into prediction models for CRC in addition to traditional risk factors. We also assessed which factors affect such improvement.

However, our study has some limitations. As previously mentioned, we identified extremely different prediction models, both in terms of genetic factors included in the models and in the methods used to include them -which range from weighted and unweighted GRS, to machine learning methods. The accuracy of a model, in terms of AUC values, depends not only on predictors that were used, but also on the method used for its construction. [64] Hence, as expected, this led to high heterogeneity of the results of our meta-analysis, which parallels what was previously described by Fung et al. regarding breast cancer [58]. Even though we showed that some factors partially explain such heterogeneity, our results should be considered exploratory and not conclusive due to the differences showed by included studies regarding chosen SNPs and traditional risk factors, as well as GRS computation methods.

Moreover, we found very limited high-quality evidence, with only one study having an overall low risk of bias [65], while majority had a high risk of bias. This not only limits the strength of our results, but also strongly suggests the need for better reporting, using as guidance the GRIPS Statement [66] or its updates, such as Polygenic Risk Score Reporting Standards (PRS-RS) [67], and higher quality research in the field of prediction models, which applies to CRC, and other chronic conditions – e.g. cardiovascular diseases [68]. Notably, all these factors affecting heterogeneity might have had an impact also on other estimates we reported in the analysis. Indeed, discriminatory accuracy of prediction models is expected to improve with the addition of newly discovered SNPs, [60] partially in contrast with our results. However, recently Khera et al. constructed 30 PRSs using millions of SNPs for five common diseases, obtaining PRSs with lower AUC values than those based on genome-wide significant SNPs only [69, 70]. This underlines the striking importance of an appropriate choice of SNPs to include in the models [58]. In addition, it should be noted that some SNPs used for risk prediction models by studies included in our analysis might have not been confirmed as risk loci by subsequent larger GWASs.

Furthermore, while recent research efforts in the field of PRS modelling are going towards the inclusion of thousand or even million SNPs into prediction models through the use of sophisticated methods, [70] such as LDpred2, lassosum, PRS-CS, and others, [71–73] the highest number of SNPs in the models included in our analyses was less than one hundred, thus limiting the applicability of our findings.

To further implement and advance knowledge in the field, in near the future, the adequate application of existing guidelines to improve the quality of prediction model studies, especially regarding study design and/or standardization of methodology to conduct these types of study, will be essential [20]. We showed that the addition of genetic factors into a prediction model with only traditional risk factors improves its performance, even if slightly. However, it is arguable if such improvement could really have an impact on populations’ health. In particular, in the field of disease prediction, great attention should be paid not only to the prediction performance, but also to clinical utility of the models [60]. As for CRC, disease prediction might play a key role in the personalization of screening programs, which could start earlier for individuals proven to be at higher risk compared with the average population. Hence, the use of a prediction model, especially if also incorporating genetic factors, might greatly impact starting age of screening [35, 74]. In addition, knowing own personal risk of cancer could also be a useful trigger for individuals to improve their adherence to screening programs, which is known to be far from the target levels [75].

The addition of genetic information may offer greater benefit when the models are used for risk prediction among specific subgroups of the population [8, 58]. This might imply that, in the future, this kinds of screening interventions could be an implemented multi-step process: the first regards the stratification of individuals according to their level of risk, followed by personalization of the interventions to carry out [58].

Eventually, as recently reported by Naber et al. [76], if a prediction model having an AUC of at least 0.65 is adopted, stratified screening for CRC becomes cost-effective compared with the current uniform screening [77]. This further underlines the importance to carry out further research in this field to improve performances of developed prediction models.

## Conclusions

The integration of genetic information into traditional prediction risk models improves the discrimination accuracy respect to CRC. However, we could not find any association or correlation respect to the number of SNPs added to the model and an AUC improvement. High heterogeneity in the choice of baseline model, method of incorporating genetic information, and studied population suggest that standardization in the conduction of this kind of studies be needed. Further steps in research are surely needed in order to improve knowledge, increase comprehension and target people who would benefit more from this intervention. It is also crucial to consider how to apply the studied models into clinical and real-life settings, in fact, the implementation of prediction models into practice will require a better comprehension of potential economic benefits and organizational effects, as well as patient safety, ethical, social, and legal implications, which will make the impact of polygenic prediction models on Health Systems clearer.

## Supplementary Information

**Additional file 1.**

**Additional file 2: Table S1.** Details of single nucleotide polymorphisms investigated by the studies included in the systematic review.

## Data Availability

All data relevant to the study are included in the published article, and can be also found in original articles included in our study.

## Abbreviations

CRC: Colorectal cancer
HDI: Human Development Index
GRS: Genetic risk score
PRS: Polygenic risk score
GWAS: Genome-wide association study
SNP: Single nucleotide polymorphism
PICO: Population, Intervention, Comparator, Outcome
PRISMA: Preferred Reporting Items for Systematic Reviews and Meta-Analyses
CHARMS: CHecklist for critical Appraisal and data extraction for systematic Reviews of prediction Modelling Studies
AUC: Area Under Curve
IDI: Integrated discrimination improvement
NRI: Net reclassification improvement
PROBAST: Prediction model Risk Of Bias ASsessment Tool

## References

[1] Bray F, Ferlay J, Soerjomataram I, Siegel RL, Torre LA, Jemal A (2018). Global cancer statistics 2018: GLOBOCAN estimates of incidence and mortality worldwide for 36 cancers in 185 countries. CA Cancer J Clin.

[2] Wong MCS, Huang J, Lok V, Wang J, Fung F, Ding H, et al. Differences in incidence and mortality trends of colorectal cancer worldwide based on sex, age, and anatomic location. Clin Gastroenterol Hepatol. 2020;0(0) [cited 2020 Sep 1]. 10.1016/j.cgh.2020.02.026. 10.1016/j.cgh.2020.02.026 32088300

[3] Gini A, Jansen EEL, Zielonke N, Meester RGS, Senore C, Anttila A (2020). Impact of colorectal cancer screening on cancer-specific mortality in Europe: a systematic review. Eur J Cancer.

[4] Zhang J, Cheng Z, Ma Y, He C, Lu Y, Zhao Y (2017). Effectiveness of screening modalities in colorectal cancer: a network meta-analysis. Clin Colorectal Cancer.

[5] Fitzpatrick-Lewis D, Ali MU, Warren R, Kenny M, Sherifali D, Raina P (2016). Screening for colorectal cancer: a systematic review and meta-analysis. Clin Colorectal Cancer.

[6] Navarro M, Nicolas A, Ferrandez A, Lanas A (2017). Colorectal cancer population screening programs worldwide in 2016: an update. World J Gastroenterol.

[7] Usher-Smith JA, Walter FM, Emery JD, Win AK, Griffin SJ (2016). Risk prediction models for colorectal cancer: a systematic review. Cancer Prev Res.

[8] McGeoch L, Saunders CL, Griffin SJ, Emery JD, Walter FM, Thompson DJ (2019). Risk prediction models for colorectal cancer incorporating common genetic variants: a systematic review. Cancer Epidemiol Biomark Prev.

[9] GWAS Catalog. Colorectal cancer. [cited 2020 Sep 3]. https://www.ebi.ac.uk/gwas/efotraits/EFO_0005842

[10] Czene K, Lichtenstein P, Hemminki K (2002). Environmental and heritable causes of cancer among 9.6 million individuals in the Swedish family-cancer database. Int J Cancer.

[11] Lichtenstein P, Holm NV, Verkasalo PK, Iliadou A, Kaprio J, Koskenvuo M (2000). Environmental and heritable factors in the causation of cancer — analyses of cohorts of twins from Sweden, Denmark, and Finland. N Engl J Med.

[12] Richardson WS, Wilson MC, Nishikawa J, Hayward RS (1995). The well-built clinical question: a key to evidence-based decisions. ACP J Club.

[13] Moher D, Liberati A, Tetzlaff J, Altman DG, Altman G (2009). Preferred reporting items for systematic reviews and meta-analyses : the PRISMA statement all use subject to JSTOR terms and conditions REPORTING items preferred for systematic reviews reporting meta-analyses : the PRISMA statement. BMJ..

[14] Moons KGM, de Groot JAH, Bouwmeester W, Vergouwe Y, Mallett S, Altman DG (2014). Critical appraisal and data extraction for systematic reviews of prediction Modelling studies: the CHARMS checklist. PLoS Med.

[15] Hanley JA, McNeil BJ (1982). The meaning and use of the area under a receiver operating characteristic (ROC) curve. Radiology.

[16] Pencina MJ, D’Agostino RB, D’Agostino RB, Vasan RS (2008). Evaluating the added predictive ability of a new marker: from area under the ROC curve to reclassification and beyond. Stat Med.

[17] Pencina MJ, D’Agostino RB, Pencina KM, Janssens ACJW, Greenland P (2012). Interpreting incremental value of markers added to risk prediction models. Am J Epidemiol.

[18] Goldman N, Glei DA (2015). Quantifying the value of biomarkers for predicting mortality. Ann Epidemiol.

[19] Yates JF (1982). External correspondence: decompositions of the mean probability score. Organ Behav Hum Perform.

[20] Wolff RF, Moons KGM, Riley RD, Whiting PF, Westwood M, Collins GS (2019). PROBAST: a tool to assess the risk of bias and applicability of prediction model studies. Ann Intern Med.

[21] StataCorp (2013). Stata statistical software: release 13.

[22] Abe M, Ito H, Oze I, Nomura M, Ogawa Y, Matsuo K (2017). The more from east-Asian, the better: risk prediction of colorectal cancer risk by GWAS-identified SNPs among Japanese. J Cancer Res Clin Oncol.

[23] Balavarca Y, Weigl K, Thomsen H, Brenner H (2020). Performance of individual and joint risk stratification by an environmental risk score and a genetic risk score in a colorectal cancer screening setting. Int J Cancer.

[24] Chandler P, Tobias D, Wang L, Smith-Warner S, Chasman D, Rose L (2018). Association between vitamin D genetic risk score and cancer risk in a large cohort of U.S. women. Nutrients.

[25] Cho YA, Lee J, Oh JH, Chang HJ, Sohn DK, Shin A (2019). Genetic risk score, combined lifestyle factors and risk of colorectal cancer. Cancer Res Treat.

[26] de Kort S, Simons CCJM, van den Brandt PA, Janssen-Heijnen MLG, Sanduleanu S, Masclee AAM (2019). Diabetes mellitus, genetic variants in the insulin-like growth factor pathway and colorectal cancer risk. Int J Cancer.

[27] Dunlop MG, Tenesa A, Farrington SM, Ballereau S, Brewster DH, Koessler T (2013). Cumulative impact of common genetic variants and other risk factors on colorectal cancer risk in 42 103 individuals. Gut.

[28] Hiraki LT, Qu C, Hutter CM, Baron JA, Berndt SI, Bézieau S (2013). Genetic predictors of circulating 25-hydroxyvitamin D and risk of colorectal cancer. Cancer Epidemiol Biomark Prev.

[29] Hosono S, Ito H, Oze I, Watanabe M, Komori K, Yatabe Y (2016). A risk prediction model for colorectal cancer using genome-wide association study-identified polymorphisms and established risk factors among Japanese. Eur J Cancer Prev.

[30] Hsu L, Jeon J, Brenner H, Gruber SB, Schoen RE, Berndt SI (2015). A model to determine colorectal cancer risk using common genetic susceptibility loci. Gastroenterology..

[31] Huyghe JR, Bien SA, Harrison TA, Kang HM, Chen S, Schmit SL (2019). Discovery of common and rare genetic risk variants for colorectal cancer. Nat Genet.

[32] Ibáñez-Sanz G, Diéz-Villanueva A, Alonso MH, Rodríguez-Moranta F, Pérez-Gómez B, Bustamante M (2017). Risk model for colorectal cancer in Spanish population using environmental and genetic factors: results from the MCC-Spain study. Sci Rep.

[33] Iwasaki M, Tanaka-Mizuno S, Kuchiba A, Yamaji T, Sawada N, Goto A (2017). Inclusion of a genetic risk score into a validated risk prediction model for colorectal cancer in Japanese men improves performance. Cancer Prev Res.

[34] Jenkins MA, Win AK, Dowty JG, MacInnis RJ, Makalic E, Schmidt DF (2019). Ability of known susceptibility SNPs to predict colorectal cancer risk for persons with and without a family history. Familial Cancer.

[35] Jeon J, Du M, Schoen RE, Hoffmeister M, Newcomb PA, Berndt SI (2018). Determining risk of colorectal cancer and starting age of screening based on lifestyle, environmental, and genetic factors. Gastroenterology..

[36] Jo J, Nam CM, Sull JW, Yun JE, Kim SY, Lee SJ (2012). Prediction of colorectal cancer risk using a genetic risk score: the Korean cancer prevention study-II (KCPS-II). Genomics Inform.

[37] Jung KJ, Won D, Jeon C, Kim S, Il KT, Jee SH (2015). A colorectal cancer prediction model using traditional and genetic risk scores in Koreans. BMC Genet.

[38] Jung SY, Zhang Z-F (2019). The effects of genetic variants related to insulin metabolism pathways and the interactions with lifestyles on colorectal cancer risk. Menopause.

[39] Marshall KW, Mohr S, Khettabi F, El Nossova N, Chao S, Bao W (2010). A blood-based biomarker panel for stratifying current risk for colorectal cancer. Int J Cancer.

[40] Prizment AE, Folsom AR, Dreyfus J, Anderson KE, Visvanathan K, Joshu CE (2013). Plasma C-reactive protein, genetic risk score, and risk of common cancers in the atherosclerosis risk in communities study. Cancer Causes Control.

[41] Rodriguez-Broadbent H, Law PJ, Sud A, Palin K, Tuupanen S, Gylfe A (2017). Mendelian randomisation implicates hyperlipidaemia as a risk factor for colorectal cancer. Int J Cancer.

[42] Schmit SL, Edlund CK, Schumacher FR, Gong J, Harrison TA, Huyghe JR (2019). Novel common genetic susceptibility loci for colorectal cancer. J Natl Cancer Inst.

[43] Shi Z, Yu H, Wu Y, Lin X, Bao Q, Jia H (2019). Systematic evaluation of cancer-specific genetic risk score for 11 types of cancer in the cancer genome atlas and electronic medical records and genomics cohorts. Cancer Med.

[44] Smith T, Gunter MJ, Tzoulaki I, Muller DC (2018). The added value of genetic information in colorectal cancer risk prediction models: development and evaluation in the UK biobank prospective cohort study. Br J Cancer.

[45] Thrift AP, Gong J, Peters U, Chang-Claude J, Rudolph A, Slattery ML (2015). Mendelian randomization study of height and risk of colorectal cancer. Int J Epidemiol.

[46] Thrift AP, Gong J, Peters U, Chang-Claude J, Rudolph A, Slattery ML (2015). Mendelian randomization study of body mass index and colorectal cancer risk. Cancer Epidemiol Biomark Prev.

[47] Wang HM, Chang TH, Lin FM, Chao TH, Huang WC, Liang C (2013). A new method for post genome-wide association study (GWAS) analysis of colorectal cancer in Taiwan. Gene..

[48] Wang K, Bai Y, Chen S, Huang J, Yuan J, Chen W (2018). Genetic correction improves prediction efficiency of serum tumor biomarkers on digestive cancer risk in the elderly Chinese cohort study. Oncotarget..

[49] Weigl K, Thomsen H, Balavarca Y, Hellwege JN, Shrubsole MJ, Brenner H (2018). Genetic risk score is associated with prevalence of advanced neoplasms in a colorectal cancer screening population. Gastroenterology..

[50] Weigl K, Chang-Claude J, Knebel P, Hsu L, Hoffmeister M, Brenner H (2018). Strongly enhanced colorectal cancer risk stratification by combining family history and genetic risk score. Clin Epidemiol.

[51] Xin J, Chu H, Ben S, Ge Y, Shao W, Zhao Y (2018). Evaluating the effect of multiple genetic risk score models on colorectal cancer risk prediction. Gene..

[52] Xin J, Du M, Gu D, Ge Y, Li S, Chu H (2019). Combinations of single nucleotide polymorphisms identified in genome-wide association studies determine risk for colorectal cancer. Int J Cancer.

[53] Yeh CC, Sung FC, Tang R, Chang-Chieh CR, Hsieh LL (2007). Association between polymorphisms of biotransformation and DNA-repair genes and risk of colorectal cancer in Taiwan. J Biomed Sci.

[54] Zhang L, Zheng C, Li T, Xing L, Zeng H, Li T, et al. Building up a robust risk mathematical platform to predict colorectal cancer. Complexity. 2017;2017.

[55] Burgess S, Scott RA, Timpson NJ, Smith GD, Thompson SG (2015). Using published data in Mendelian randomization: a blueprint for efficient identification of causal risk factors. Eur J Epidemiol.

[56] Swets JA (1988). Measuring the accuracy of diagnostic systems. Science.

[57] Han M, Choong TL, Hong WZ, Chao S, Zheng R, Kok TY (2008). Novel blood-based, five-gene biomarker set for the detection of colorectal cancer. Clin Cancer Res.

[58] Fung SM, Wong XY, Lee SX, Miao H, Hartman M, Wee HL (2019). Performance of single-nucleotide polymorphisms in breast cancer risk prediction models: a systematic review and meta-analysis. Cancer Epidemiol Biomark Prev.

[59] Cook NR (2018). Quantifying the added value of new biomarkers: how and how not. Diagnostic Progn Res.

[60] Cecile A, Janssens JW, Joyner MJ (2019). Polygenic risk scores that predict common diseases using millions of single nucleotide polymorphisms: is more, better?. Clin Chem.

[61] Tzoulaki I, Liberopoulos G, Ioannidis JPA (2009). Assessment of claims of improved prediction beyond the Framingham risk score. JAMA - J Am Med Assoc.

[62] Martin AR, Kanai M, Kamatani Y, Okada Y, Neale BM, Daly MJ (2019). Clinical use of current polygenic risk scores may exacerbate health disparities. Nat Genet.

[63] Marigorta UM, Rodríguez JA, Gibson G, Navarro A (2018). Replicability and prediction: lessons and challenges from GWAS. Trends Genet.

[64] Kundu S, Mihaescu R, CMC M, Bakker R, Janssens ACJW. Estimating the predictive ability of genetic risk models in simulated data based on published results from genome-wide association studies. Front Genet. 2014;5(JUN) [cited 2020 Nov 17]. https://pubmed.ncbi.nlm.nih.gov/24982668/. 10.3389/fgene.2014.00179 PMC4056181 24982668

[65] Luo H, Zhao Q, Wei W, Zheng L, Yi S, Li G, et al. Circulating tumor DNA methylation profiles enable early diagnosis, prognosis prediction, and screening for colorectal cancer. Sci Transl Med. 2020;12(524) [cited 2020 Aug 26]. http://stm.sciencemag.org/. 10.1126/scitranslmed.aax7533 31894106

[66] Janssens ACJW, Ioannidis JPA, van Duijn CM, Little J, Khoury MJ (2011). Strengthening the reporting of genetic risk prediction studies: the GRIPS statement. PLoS Med.

[67] Wand H, Lambert SA, Tamburro C, Iacocca MA, O’Sullivan JW, Sillari C (2021). Improving reporting standards for polygenic scores in risk prediction studies. Nature..

[68] Fiatal S, Ádány R (2018). Application of single-nucleotide polymorphism-related risk estimates in identification of increased genetic susceptibility to cardiovascular diseases: a literature review. Front Public Health.

[69] Janssens ACJW. Validity of polygenic risk scores: are we measuring what we think we are?. 28, Hum Mol Genet. 2019;R143–R150. Oxford University Press; [cited 2020 Nov 17]. https://academic.oup.com/hmg/article/28/R2/R143/5555564 10.1093/hmg/ddz205 PMC7013150 31504522

[70] Khera AV, Chaffin M, Aragam KG, Haas ME, Roselli C, Choi SH (2018). Genome-wide polygenic scores for common diseases identify individuals with risk equivalent to monogenic mutations. Nat Genet.

[71] Privé F, Arbel J, Vilhjálmsson BJ. LDpred2: better, faster, stronger. Schwartz R, editor. Bioinformatics. 2020; [cited 2021 Mar 18]; https://academic.oup.com/bioinformatics/advance-article/doi/10.1093/bioinformatics/btaa1029/6039173. 10.1093/bioinformatics/btaa1029 PMC8016455 33326037

[72] Mak TSH, Porsch RM, Choi SW, Zhou X, Sham PC (2017). Polygenic scores via penalized regression on summary statistics. Genet Epidemiol.

[73] Ge T, Chen CY, Ni Y, Feng YCA, Smoller JW (2019). Polygenic prediction via Bayesian regression and continuous shrinkage priors. Nat Commun.

[74] Kuipers EJ, Spaander MC (2018). Personalized screening for colorectal cancer. Nat Rev Gastroenterol Hepatol.

[75] Robertson DJ, Ladabaum U (2019). Opportunities and challenges in moving from current guidelines to personalized colorectal cancer screening. Gastroenterology.

[76] Naber SK, Kundu S, Kuntz KM, Dotson WD, Williams MS, Zauber AG, et al. Cost-effectiveness of risk-stratified colorectal cancer screening based on polygenic risk: current status and future potential. JNCI Cancer Spectr. 2020;4(1) [cited 2020 Aug 26]. https://academic.oup.com/jncics/article/4/1/pkz086/5586982. 10.1093/jncics/pkz086 PMC6988584 32025627

[77] Bibbins-Domingo K, Grossman DC, Curry SJ, Davidson KW, Epling JW, García FAR (2016). Screening for colorectal cancer: US preventive services task force recommendation statement. JAMA-J Am Med Assoc.

