# Supplementary material for: Polygenic risk prediction models for colorectal cancer: a systematic review
Source: BMC Cancer. 2022 Jan 15;22:65. doi: 10.1186/s12885-021-09143-2 (PMC8760647; doi:10.1186/s12885-021-09143-2)
Supplement: Supplementary file 2 — Additional file 2: Table S1. Details of single nucleotide polymorphisms investigated by the studies included in the systematic review. [file 12885_2021_9143_MOESM2_ESM.pdf]

Table S1. Details of single nucleotide polymorphisms investigated by the studies included in the systematic review.

First author, year (ref)	SNPs
Abe M, 2017 (22)	US/Europeans: rs6983267, rs4779584, rs4444235, rs9929218.<br><br>East Asians: rs10936599, rs16969681, rs704017, rs11196172, rs10774214, rs647161, rs2423279.
Balavarca Y, 2019 (23)	rs72647484, rs10911251, rs6687758, rs6691170, rs11903757, rs812481, rs35360328, rs10936599, rs3987, rs35509282, rs647161, rs1321311, rs11986063, rs16892766, rs7837328, rs6983267, rs10505477, rs7014346, rs719725, rs10904849, rs10795668, rs1035209, rs11190164, rs3824999, rs3802842, rs11213809, rs10774214, rs3217810, rs3217901, rs11169552, rs7136702, rs3184504, rs59336, rs73208120, rs1957636, rs4444235, rs17094983, rs11632715, rs16969681, rs4779584, rs9929218, rs1862748, rs16941835, rs4939827, rs12953717, rs4464148, rs10411210, rs7259371, rs2423279, rs4813802, rs961253, rs355527, rs6066825, rs4925386, rs5934683.
Chandler PD, 2018 (51)	rs11234027, rs7944926, rs12794714, rs10741657, rs2282679.
Cho YA, 2019 (24)	rs647161, rs6983267, rs7014346, rs10505477, rs10795668, rs704017, rs11196172, rs174537, rs174550, rs1535, rs4779584, rs10411210, rs2423279.
deKort S, 2019 (53)	rs1520220, rs5742678, rs5742694, rs10735380, rs2854744, rs2132572, rs35440925, rs1801278, rs1805097, rs2289046, rs754204, rs4773082, rs4988496, rs1801282, rs1501299, rs2241766, rs266729, rs1648707, rs182052, rs1342387, rs7539542, rs12733285, rs1044471, rs767870.
Dunlop MG, 2013 (25)	rs6983267, rs4779584, rs4939827, rs3802842, rs10795668, rs16892766, rs4444235, rs9929218, rs10411210, rs961253.
Hiraki LT, 2013 (26)	rs2282679, rs10741657, rs12785878, rs6013897.
Hosono S, 2016(27)	rs6983267, rs4779584, rs1696961, rs444435, rs9929218, rs1093599.
HsuL, 2015(28)	rs16892766, rs6983267, rs10795668, rs3802842, rs4444235, rs4779584, rs9929218, rs4939827, rs10411210, rs961253, rs6687758, rs10936599, rs1321311, rs719725, rs3824999, rs7136702, rs1957636, rs4813802, rs4925386, rs10911251, rs11903757, rs3217810, rs3217901, rs59336, rs647161, rs10774214, rs2423279.
Huyghe JR, 2019(29)	rs72647484, rs4360494, rs12144319, rs6678517, rs17011141, rs448513, rs11884596, rs983402, rs3731861, rs35470271, rs6781752, rs72942485, rs10049390, rs9876206, rs13149359, rs1391441, rs11727676, rs78368589, rs2735940, rs7708610, rs12514517, rs145364999, rs755229494, rs4976270, rs2516420, rs9271695, rs16878812, rs9470361, rs62396735, rs62404966, rs12672022, rs16892766, rs6469654, rs117079142, rs6983267, rs4313119, rs1537372, rs34405347, rs10980628, rs11255841, rs10821907, rs704017, rs11190164, rs12246635, rs11196170, rs174533, rs7121958, rs61389091, rs2186607, rs3087967, rs35808169, rs3217810, rs3217874, rs2250430, rs2710310, rs11610543, rs12372718, rs4759277, rs597808, rs7300312, rs377429877, rs7333607, rs78341008, rs8000189, rs35107139, rs4901473, rs17094983, rs12708491, rs2293581, rs17816465, rs56324967, rs9924886, rs9930005, rs12149163, rs62042090, rs4968127, rs1078643, rs983318, rs75954926, rs11874392, rs34797592, rs28840750, rs1963413, rs73068325, rs189583, rs994308, rs4813802, rs28488, rs11087784, rs6058093, rs6031311, rs6066825, rs6063514, rs1741640, rs2738783.
Ibáñez-Sanz G, 2017(30)	rs10752881, rs6691170, rs10936599, rs1321311, rs7758229, rs16892766, rs6983267, rs10795668, rs4948317, rs3802842, rs3824999, rs10879357,

	rs11169552, rs7315438, rs4444235, rs9929218, rs4939827, rs10411210, rs4925386, rs961253, rs5934683.
Iwasaki M, 2017(31)	rs6983267, rs3802842, rs1035209, rs12241008, rs174537, rs49398276.
Jenkins MA, 2019(32)	rs72647484, rs10911251, rs6687758, rs11903757, rs812481, rs35360328, rs10936599, rs3987, rs35509282, rs647161, rs1321311, rs16892766, rs6983267, rs719725, rs10904849, rs10795668, rs704017, rs11190164, rs12241008, VTI1A; 11qhap, rs3824999, rs3802842, rs3217810, rs3217901, rs10774214, rs11169552, rs7136702, rs3184504, rs59336, rs73208120, rs1957636, rs4444235, rs11632715, rs16969681, rs9929218, rs16941835, rs744166, rs4939827, rs10411210, 19qhap, rs2423279, rs4813802, rs961253, rs6066825, rs4925386.
Jeon J, 2018(33)	rs10911251, rs6687758, rs6691170, rs72647484, rs11903757, rs812481, rs35360328, rs10936599, rs647161, rs202110856c, rs1321311, rs4711689, rs7758229, rs16892766, rs2450115, rs6469656, rs10505477, rs6983267, rs7014346, rs719725, rs10904849, rs10795668, rs704017, rs1035209, rs11190164, rs4919687, rs12241008, rs10506868, rs11196172, rs1535, rs174537, rs174550, rs4246215, rs3824999, rs3802842, rs10849432, rs3217810, rs10774214, rs11064437, rs11169552, rs7136702, rs3184504, rs73208120, rs1957636, rs4444235, rs4779584, rs11632715, rs16969681, rs79900961, rs9929218, rs16941835, rs12603526, rs4939827, rs7229639, rs10411210, rs1800469, rs2241714, rs2423279, rs4813802, rs961253, rs6066825, rs4925386, rs6061231.
Jo J, 2012(34)	Men: rs17391002, rs9549448, rs254833.<br><br>Women: rs10083736, rs16987827, rs8046516, rs9926182, rs17523778.
Jung KJ, 2015(54)	rs3802842, rs4939827, rs6983267, rs10505477, rs10795668, rs961253, rs9929218.
Jung SY, 2019(48)	rs10778176, rs10860865, rs1520220, rs5742671, rs6214, rs7136446, rs978458, rs2471551, rs3110697, rs3842763, rs689, rs1801123, rs1801278, rs1130214, rs2494738, rs2494740, rs2494744, rs2498789, rs11673367, rs2304186, rs3730256, rs4332845, rs7247515, rs13058, rs17759796, rs17821572, rs2266966, rs2283791, rs2298432, rs5999550, rs7286558, rs743411, rs9610496, rs9610505, rs1043526, rs10515070, rs12657050, rs1445760, rs1664577, rs16897558, rs171649, rs173702, rs173703, rs1862162, rs251399, rs251404, rs251406, rs34306, rs3730089, rs706711, rs7707370, rs831123, rs831125, rs11196205.
Prizment AE, 2013(52)	rs4420638, rs2794520, rs1183910, rs10521222, rs4420065, rs1800961, rs4129267, rs1260326, rs9987289, rs13233571, rs6734238, rs12239046, rs12037222, rs4705952, rs10745954, rs6901250, rs2836878, rs4903031, rs340029, rs2847281.
Rodriguez-Broadbent H, 2017(36)	Total Cholesterol: rs1077514, rs2287623, rs11694172, rs11563251, rs13315871 rs2758886, rs9376090, rs1997243, rs3780181, rs10904908 rs11603023, rs4883201, rs314253, rs138777, rs4253772, rs12027135 rs7515577, rs2642442, rs514230, rs7570971, rs2290159, rs12916, rs6882076, rs3177928, rs2814982, rs9488822 rs12670798, rs2072183, rs2081687, rs2255141, rs10128711 rs11065987, rs1169288, rs2000999, rs10401969, rs492602, rs2277862, rs2902940.<br><br>Triglyceride: rs38855, rs1832007, rs3198697, rs8077889, rs7248104, rs645040, rs442177, rs9686661, rs11776767, rs10761731, rs2068888, rs2412710, rs11649653, rs5756931.<br><br>LDL: rs267733, rs2710642, rs1250229, rs4942486, rs1801689, rs364585, rs2328223, rs5763662, rs8017377.<br><br>HDL: rs12145743, rs4650994, rs1047891, rs2606736, rs2290547, rs2013208, rs13326165, rs6805251, rs10019888, rs3822072, rs2602836, rs702485, rs4142995,

	rs4917014, rs17173637, rs12801636, rs499974, rs4983559, rs17695224, rs4660293, rs1689800, rs12328675, rs13107325, rs6450176, rs605066, rs4731702, rs2293889, rs2923084, rs3136441, rs7134375, rs7255436, rs737337, rs386000.
Schmit SL, 2019(37)	rs10911251, rs6691170, rs6687758, rs11903757, rs992157, rs35360328, rs812481, rs10936599, rs1370821, rs3987, rs35509282, rs2735940, rs58791712, rs647161, rs6906359, rs1321311, rs4711689, rs62404968, rs7758229, rs140355816, rs2450115, rs16892766, rs6469656, rs10505477, rs6983267, rs7014346, rs719725, rs10795668, rs11255841, rs10994860, rs704017, rs1035209, rs11190164, rs4919687, rs12241008, rs10506868, rs11196172, rs174537, rs4246215, rs174550, rs1535, rs3824999, rs3802842, rs10774214, rs3217810, rs3217901, rs10849432, rs34245511, rs11169552, rs3184504, rs59336, rs72013726, rs73208120, rs4444235, rs1957636, rs17094983, rs16969681, rs4779584, rs11632715, rs73376930, rs9929218, rs2696839, rs12603526, rs7229639, rs4939827, rs10411210, rs1800469, rs2241714, rs961253, rs4813802, rs2423279, rs6066825, rs1810502, rs4925386, rs6061231, rs2427308.
Shi Z, 2019(38)	rs10911251, rs6691170, rs6687758, rs11903757, rs992157, rs1370821, rs6906359, rs1321311, rs62404968, rs16892766, rs6983267, rs10994860, rs1035209, rs1535, rs3824999, rs3802842, rs7136702, rs11169552, rs3184504, rs73208120, rs1957636, rs17094983, rs4779584, rs1862748, rs4939827, rs10411210, rs355527, rs8124813, rs1810502, rs4925386.
Smith T, 2018(49)	rs10911251, rs6687758, rs11903757, rs812481, rs35360328, rs10936599, rs647161, rs1321311, rs7758229, rs16892766, rs6983267, rs719725, rs10795668, rs704017, rs1035209, rs12241008, rs11196172, rs1535, rs3824999, rs3802842, rs10849432, rs3217810, rs10774214, rs11169552, rs7136702, rs3184504, rs73208120, rs1957636, rs4444235, rs4779584, rs9929218, rs12603526, rs4939827, rs7229639, rs10411210, rs1800469, rs2423279, rs4813802, rs961253, rs6066825, rs4925386.
Thrift AP, 2015(39)	rs425277, rs9434723, rs10779751, rs2284746, rs12137162, rs212524, rs1014987, rs2806561, rs4601530, rs926438, rs17163588, rs2219320, rs12119525, rs16834765, rs7544462, rs209918, rs6600365, rs3014219, rs564914, rs12855, rs17387330, rs6691924, rs2815379, rs12731056, rs17391694, rs567401, rs2046158, rs7551732, rs2811594, rs17113369, rs7517682, rs12120956, rs1321666, rs9428104, rs1409156, rs12144094, rs6658763, rs7534365, rs3767627, rs956796, rs2298265, rs6688100, rs4656220, rs6694089, rs12125882, rs2421992, rs17369123, rs1325596, rs4652773, rs3814333, rs2275325, rs10863936, rs6540834, rs1244981, rs10495098, rs991967, rs12411277, rs4428898, rs1935157, rs1544196, rs6696239, rs11799609, rs17038954, rs10048625, rs3885668, rs2345835, rs13006748, rs7561273, rs2278483, rs2289195, rs10460566, rs780094, rs7605699, rs6714546, rs6751657, rs711245, rs6544089, rs17511102, rs12615742, rs13416119, rs9309101, rs897080, rs17032525, rs12474201, rs354196, rs1367226, rs3791679, rs3791673, rs2120335, rs7568069, rs11684404, rs11683207, rs13388725, rs2166898, rs7567288, rs4953951, rs749234, rs540652, rs12987566, rs6746356, rs7567851, rs833152, rs12693589, rs6435143, rs4425077, rs12329133, rs17181956, rs994533, rs1864439, rs992157, rs2305833, rs4674354, rs12470505, rs16859517, rs12621643, rs6761041, rs6733349, rs6754426, rs4973429, rs2679184, rs749052, rs11677466, rs3116168, rs2343240, rs13393800, rs4344931, rs11687941, rs2633761, rs13078528, rs2596831, rs2597513, rs11708412, rs9816693, rs3915129, rs13088462, rs4256170, rs2240919, rs2581830, rs2034172, rs9835332, rs1658351, rs6794009, rs17806888, rs2175513, rs12330322, rs7633464, rs9825951, rs1797625, rs1533269, rs1546391, rs6439168, rs4974480, rs6762606, rs9880211, rs724016, rs11714558, rs936339, rs4325879, rs6441170, rs7652177, rs509035, rs9858528, rs16860216, rs720390, rs2300921, rs4686904, rs7646824, rs9841435, rs3958122, rs867245, rs11722554,

	rs6446315, rs868489, rs6829680, rs2302580, rs763318, rs4834927, rs7692995, rs961014, rs16994718, rs2306596, rs1996422, rs13113518, rs17081935, rs9993613, rs17556750, rs2167645, rs11731978, rs6813055, rs12639764, rs1562975, rs7659107, rs6838153, rs12513181, rs11100790, rs7654571, rs6845999, rs1812175, rs17777628, rs4240326, rs996743, rs13133465, rs13150868, rs955748, rs17410035, rs7731703, rs3811958, rs9292468, rs13183624, rs11745439, rs1004202, rs301901, rs3812040, rs17574650, rs2961830, rs7716219, rs2662027, rs7727731, rs10059761, rs9291926, rs34651, rs820848, rs12519505, rs7712162, rs32855, rs6894139, rs2247870, rs12186664, rs6594336, rs13177718, rs1582931, rs6887276, rs26024, rs39623, rs7701414, rs6596075, rs526896, rs9327705, rs165189, rs4624820, rs2974438, rs4620037, rs1529701, rs33852, rs12153391, rs4868126, rs1368380, rs17075869, rs7733195, rs4868645, rs422421, rs11950938, rs12055154, rs11750568, rs6879260, rs932445, rs163071, rs4246079, rs17603945, rs9392918, rs9328445, rs9405356, rs17330192, rs1047014, rs4141885, rs806794, rs1233627, rs9404952, rs1265097, rs6457374, rs2857693, rs3129254, rs12204421, rs3957165, rs12214804, rs3800461, rs6919534, rs6899744, rs4713902, rs16895130, rs1040941, rs10948222, rs9395264, rs12190423, rs12209223, rs6903448, rs648831, rs1341278, rs9443804, rs310421, rs3828760, rs761391, rs314263, rs479744, rs6920372, rs2145357, rs1405212, rs389663, rs4895801, rs1155939, rs1415701, rs7740107, rs6921207, rs7743622, rs7745166, rs4896582, rs6911389, rs2748483, rs6902771, rs3020418, rs11156098, rs1832871, rs9456307, rs486359, rs991946, rs2763273, rs7774834, rs798497, rs4725061, rs929637, rs17140875, rs2390151, rs3807931, rs7782764, rs12538407, rs1055144, rs552707, rs12533079, rs6462432, rs6974574, rs1007358, rs6949739, rs2715094, rs1113765, rs12669267, rs17807185, rs2888877, rs42039, rs2188177, rs6971575, rs17250196, rs6952113, rs6962887, rs273945, rs822531, rs6955948, rs4875421, rs429433, rs7834383, rs7823327, rs4273857, rs17088184, rs2013265, rs3812423, rs568610, rs6988484, rs6999671, rs10958476, rs9650315, rs2956605, rs16939034, rs4735677, rs7007200, rs2737220, rs1550162, rs1599473, rs10283100, rs11779459, rs8180991, rs4733724, rs894343, rs1036821, rs6577717, rs11783655, rs7033940, rs2149163, rs3927536, rs10962832, rs1576900, rs3763631, rs10972628, rs11144688, rs958225, rs7853235, rs181338, rs10780910, rs1571892, rs7043114, rs1257763, rs12347744, rs4448343, rs1329393, rs817300, rs10990303, rs7870753, rs953199, rs989393, rs10820814, rs9409082, rs902143, rs2451948, rs7027110, rs3739707, rs999599, rs10759774, rs10119624, rs12344396, rs7033487, rs1742829, rs10817960, rs7466269, rs3132297, rs7849585, rs3812591, rs4332428, rs12779328, rs4350272, rs7069985, rs10995319, rs1171615, rs10997979, rs4746769, rs1815314, rs703985, rs1923367, rs2631676, rs915506, rs11599750, rs10883563, rs7899004, rs6584575, rs11198820, rs291979, rs1614303, rs7097701, rs10794175, rs11245515, rs11244750, rs2272566, rs2735469, rs4320932, rs17659078, rs2237886, rs6485978, rs2099745, rs10766065, rs7126398, rs2915404, rs757081, rs10767838, rs3802758, rs1681630, rs1945237, rs3782089, rs7112925, rs2510396, rs3750972, rs4357716, rs2509133, rs11236294, rs606452, rs632124, rs10790381, rs1461503, rs11221442, rs11612228, rs7299326, rs2856321, rs1420023, rs12228415, rs4326884, rs10770705, rs11047239, rs1861908, rs10492364, rs11049611, rs12820411, rs10843390, rs10880969, rs2306694, rs10877030, rs17122659, rs2164968, rs8756, rs11175992, rs10748128, rs17783015, rs11107062, rs3825199, rs10859567, rs7971536, rs833706, rs2164747, rs2888893, rs11616067, rs4767473, rs497273, rs11835818, rs7980687, rs11057552, rs1809889, rs1199734, rs11618507, rs12323101, rs12863103, rs6561319, rs12871822,

	rs7334755, rs2687950, rs1753637, rs3118905, rs4883972, rs3818416, rs11616380, rs6563199, rs7319045, rs7985356, rs17792664, rs8017130, rs1950500, rs12435366, rs10131337, rs6571772, rs4901537, rs8006657, rs11624136, rs2093210, rs2781373, rs1980850, rs2058092, rs862034, rs10140101, rs7154721, rs1190545, rs12882130, rs10152739, rs316618, rs1036477, rs10744956, rs16964211, rs782930, rs7177711, rs7162825, rs17264185, rs731874, rs10152591, rs975210, rs11634405, rs12904334, rs4337252, rs5742915, rs16968242, rs12914466, rs17349981, rs2257011, rs7162542, rs11855014, rs1348002, rs11633371, rs16942341, rs2280470, rs3817428, rs2238300, rs8028843, rs7181724, rs2871865, rs2573625, rs4246302, rs4548838, rs7170986, rs8042424, rs11648796, rs12597498, rs26868, rs2014467, rs12926008, rs1053996, rs129963, rs2531992, rs960006, rs1659127, rs2023693, rs11642612, rs4785393, rs9929889, rs8058684, rs1966913, rs3790086, rs217181, rs11640018, rs4243206, rs6420435, rs2326458, rs4843367, rs300039, rs3748394, rs8052560, rs2377058, rs11861084, rs870183, rs9217, rs8073177, rs1625895, rs8067165, rs8069300, rs4640244, rs3809790, rs9889755, rs3760318, rs2028067, rs2338115, rs584828, rs3169906, rs9766, rs4986172, rs199515, rs8073371, rs6504389, rs318095, rs2072153, rs4605213, rs11867943, rs227724, rs1401795, rs2079795, rs2378870, rs2044124, rs2854207, rs2070776, rs3923086, rs2072268, rs11867479, rs10083886, rs2117563, rs1552173, rs1478610, rs4239020, rs888403, rs692964, rs14062, rs4369779, rs11661645, rs12454567, rs2337143, rs12458127, rs9967417, rs11152213, rs8097893, rs11659752, rs11880992, rs2074977, rs2123731, rs891088, rs1346490, rs4072910, rs6511689, rs8102380, rs7259684, rs8103068, rs10401193, rs8103992, rs7253628, rs4802134, rs4803468, rs11880124, rs2682587, rs2059877, rs7273787, rs17721822, rs1884897, rs6085662, rs6080830, rs7261425, rs8117259, rs6137287, rs1074683, rs1535466, rs143384, rs2425163, rs4812586, rs2224538, rs17450430, rs6020202, rs1326023, rs2057291, rs3026499, rs6061231, rs2829941, rs2834442, rs2211866, rs9977276, rs2413143, rs7284476, rs5757318, rs738288, rs11090631.
Thrift AP, 2015(40)	BMI: rs1558902, rs6567160, rs13021737, rs10938397, rs543874, rs2207139, rs11030104, rs3101336, rs7138803, rs10182181, rs3888190, rs1516725, rs12446632, rs2287019, rs16951275, rs3817334, rs2112347, rs12566985, rs3810291, rs7141420, rs13078960, rs10968576, rs17024393, rs657452, rs12429545, rs12286929, rs13107325, rs11165643, rs7903146, rs10132280, rs17405819, rs1016287, rs4256980, rs17094222, rs12401738, rs7599312, rs2365389, rs205262, rs2820292, rs12885454, rs12016871, rs16851483, rs1167827, rs758747, rs1928295, rs9925964, rs11126666, rs2650492, rs6804842, rs12940622, rs11847697, rs4740619, rs13191362, rs3736485, rs17001654, rs11191560, rs1528435, rs2075650, rs1000940, rs2033529, rs11583200, rs9400239, rs10733682, rs11688816, rs11057405, rs2121279, rs29941, rs11727676, rs3849570, rs6477694, rs7899106, rs2176598, rs2245368, rs17724992, rs7243357, rs1808579, rs2033732.<br><br>Waist-hip ratio: NR
Wang HM, 2013(41)	rs4072037, rs1800872, rs231775, rs3731055, rs11721827, rs2736100, rs160277, rs1983891, rs2860580, rs712221, rs157935, rs1512268, rs3214050, rs6983267, rs1412829, rs1503185, rs869736, rs1946518, rs7975232, rs1572072, rs1760944, rs4784227, rs4430796, rs3135967, rs10411210, rs1799782.
Wang K, 2018(50)	AFP: rs12506899, rs2251844.<br><br>CA19-9: rs17271883, rs265548, rs1047781.

	CEA: rs8176749, rs8176720, rs441810.
Weigl K, 2018(42)	rs72647484, rs10911251, rs6687758, rs6691170, rs11903757, rs812481, rs35360328, rs10936599, rs3987, rs35509282, rs647161, rs1321311, rs11986063, rs16892766, rs6983267, rs719725, rs10904849, rs10795668, rs1035209, rs11190164, rs3824999, rs3802842, rs11213809, rs10774214, rs3217810, rs3217901, rs11169552, rs7136702, rs3184504, rs59336, rs73208120, rs1957636, rs4444235, rs17094983, rs11632715, rs16969681, rs4779584, rs9929218, rs16941835, rs4939827, rs12953717, rs4464148, rs10411210, rs2423279, rs4813802, rs355527, rs4925386, rs5934683.
Weigl K, 2018(43)	1p36.12, 1q25.3, 1q41, 2q32.3, 3p14.1, 3p22.1, 3q26.2, 4q26, 4q32.2, 5q31.1, 6p21.31, 8q23.3, 8q24, 9p24, 10p13, 10p14, 10q24.2, 10q24.2, 11q13.4, 11q23, 11q23.1, 12p13.32, 12p13.32, 12p13.32, 12q13.13, 12q24.12, 12q24.21, 12q24.22, 14q22.2, 14q22.2, 14q23.1, 15q13, 15q13, 15q13.3, 16q22.1, 16q24.1, 18q21.1, 18q21, 19q13.1, 20p12.3, 20p12.3, 20p12.3, 20q13.1, 20q13.33.
Xin J, 2018(44)	rs4711689, rs2450115, rs12241008, rs10506868, rs11196172, rs3824999, rs3802842, rs2238126, rs11169552, rs4779584, rs12603526, rs7229639, rs2423279, rs6066825.
Xin J, 2019(45)	Chinese individuals: rs7528276, rs10911251, rs6687758, rs1367374, rs11903757, rs992157, rs35360328, rs812481, rs10936599, rs1370821, rs35509282, rs125205347, rs12522693, rs6906359, rs1321311, rs4711689, rs62404968, rs7758229, rs2450115, rs6983267, rs719725, rs10795668, rs10994860, rs704017, rs1035209, rs4919687, rs10506868, rs11196172, rs1535, rs3824999, rs3802842, rs3217901, rs11064437, rs2238126, rs11169552, rs128229847, rs4444235, rs1957636, rs16969681, rs4779584, rs11632715, rs142319636, rs9929218, rs847208, rs2696839, rs16941835, rs7229639, rs4939827, rs4464148, rs10411210, rs1800469, rs961253, rs4813802, rs2423279, rs6065668, rs1810502, rs4925386, rs6061231.<br><br>European individuals: rs72647484, rs7528276, rs10911251, rs6691170, rs6687758, rs1367374, rs11903757, rs992157, rs35360328, rs812481, rs10936599, rs1370821, rs35509282, rs2735940, rs125205345, rs12522693, rs647161, rs1440375975, rs1321311, rs4711689, rs62404968, rs7758229, rs2450115, rs16892766, rs6469656, rs10505477, rs719725, rs10795668, rs10994860, rs704017, rs1035209, rs4919687, rs12241008, rs10506868, rs11196172, rs1665650, rs174537, rs3824999, rs3802842, rs10774214, rs3217810, rs3217901, rs10849432, rs2238126, rs7136702, rs11169552, rs3184504, rs59336, rs128229845, rs73208120, rs4444235, rs1957636, rs17094983, rs16969681, rs4779584, rs11632715, rs142319636, rs9929218, rs847208, rs2696839, rs16941835, rs12603526, rs17836917, rs7229639, rs4939827, rs4464148, rs10411210, rs1800469, rs961253, rs4813802, rs2423279, rs6065668, rs6066825, rs1810502, rs4925386.
Yeh CC, 2007 (46)	CYP1A2*1C, CYP1A1*2C, GSTM1 (null *vs* present), GSTT1 (null *vs* present), XPD Lys751Gln, XRCC3 Thr241Met, GSTP1 Ile105Val, NAT1 (rapid *vs* slow), NAT2 (medium+rapid *vs* slow), XRCC1 Arg399Gln.
Zhang L, 2017 (47)	rs10046, rs1256030, rs6766387, rs6983267.

NR: not reported/not retrieved; BMI: body mass index; SNP: single nucleotide polymorphism.
